# Dynamic Effective Connectivity Patterns During Rapid Face Stimuli Presentation in Body Dysmorphic Disorder

**DOI:** 10.3389/fnins.2022.890424

**Published:** 2022-05-24

**Authors:** Wan-wa Wong, D. Rangaprakash, Teena D. Moody, Jamie D. Feusner

**Affiliations:** ^1^Centre for Addiction and Mental Health, Toronto, ON, Canada; ^2^Athinoula A. Martinos Center for Biomedical Imaging, Massachusetts General Hospital, Harvard Medical School, Charlestown, MA, United States; ^3^Department of Psychiatry and Biobehavioral Sciences, David Geffen School of Medicine, University of California, Los Angeles, Los Angeles, CA, United States; ^4^Department of Psychiatry, Division of Neurosciences & Clinical Translation, Temerty Faculty of Medicine, University of Toronto, Toronto, ON, Canada; ^5^Department of Women’s and Children’s Health, Karolinska Institutet, Stockholm, Sweden

**Keywords:** BDD, time-varying effective connectivity, face processing, rapid face presentation, dorsal and ventral visual streams, functional MRI

## Abstract

In individuals with body dysmorphic disorder (BDD), perceptual appearance distortions may be related to imbalances in global vs. local visual processing. Understanding the mechanistic brain effects of potential interventions is crucial for rational treatment development. The dorsal visual stream (DVS) is tuned to rapid image presentation, facilitating global/holistic processing, whereas the ventral visual stream (VVS), responsible for local/detailed processing, reduces activation magnitude with shorter stimulus duration. This study tested a strategy of rapid, short-duration face presentation on visual system connectivity. Thirty-eight unmedicated adults with BDD and 29 healthy controls viewed photographs of their faces for short (125 ms, 250 ms, 500 ms) and long (3000 ms) durations during fMRI scan. Dynamic effective connectivity in DVS and VVS was analyzed. BDD individuals exhibited weaker connectivity from occipital to parietal DVS areas than controls for all stimuli durations. Short compared with long viewing durations (125 ms vs. 3,000 ms and 500 ms vs. 3,000 ms) resulted in significantly weaker VVS connectivity from calcarine cortex to inferior occipital gyri in controls; however, there was only a trend for similar results in BDD. The DVS to VVS ratio, representing a balance between global and local processing, incrementally increased with shorter viewing durations in BDD, although it was not statistically significant. In sum, visual systems in those with BDD are not as responsive as in controls to rapid face presentation. Whether rapid face presentation could reduce connectivity in visual systems responsible for local/detailed processing in BDD may necessitate different parameters or strategies. These results provide mechanistic insights for perceptual retraining treatment designs.

## Introduction

Individuals with body dysmorphic disorder (BDD) are preoccupied with misperceived appearance defects that are not noticeable to others, which sufferers believe render them ugly and deformed, causing significant distress and functional impairment (American Psychiatric Association, 2013). The body parts that are commonly misperceived involve the face and head, although any appearance feature can be of concern (Phillips, 2005). This disorder can result in profound consequences, with high lifetime prevalence of suicide attempts (25%) (Phillips and Menard, 2006) and hospitalization (50%) (Phillips et al., 1994). Twenty-seven to 39% are delusional in their beliefs (Phillips, 2004). BDD is still misdiagnosed and understudied, although it has a high point prevalence of 1.7∼2.9% in the general population (Rief et al., 2006; Koran et al., 2008; Buhlmann et al., 2010; Schieber et al., 2015) and 13% in psychiatric inpatients (Mufaddel et al., 2013). Although some neurobiological models to explain vulnerability to BDD have been discovered (Li et al., 2013; Grace et al., 2017), a comprehensive understanding of this condition is still ongoing.

In BDD, aberrant visual information processing may be a core neurobiological contributor to the psychopathology of appearance perception distortions (Li et al., 2013; Beilharz et al., 2017). From our previous neuroimaging studies using own-face (Feusner et al., 2010b), other-face (Feusner et al., 2007; Li et al., 2015a; Moody et al., 2015), house (Feusner et al., 2011; Li et al., 2015a), and body stimuli (Morfini et al., 2017; Moody et al., 2020), abnormally reduced activity and/or connectivity in the dorsal visual stream (DVS) were found when viewing images that were filtered to selectively convey configural and holistic information. This led to the hypothesis that BDD may be driven by under-utilization of brain systems dedicated to the global/holistic visual processing. This hypothesis is further supported by subsequent imaging and electro-cortical findings (Li et al., 2015a,b), showing that enhanced ventral visual stream (VVS) for local/detailed visual processing was found in BDD, and the degree of local/detailed visual processing was directly associated with how unattractive they perceived a face to be (Li et al., 2015a). These may reflect an imbalance in global and local processing.

This model of imbalance in global vs. local processing is also confirmed by neuropsychological and psychophysical studies testing face and body inversion effects (Deckersbach et al., 2000; Stangier et al., 2008; Feusner et al., 2010a; Jefferies et al., 2012; Mundy and Sadusky, 2014; Dhir et al., 2018). The face inversion effect (FIE), which is represented by lower recognition accuracy and slower performance when viewing upside-down others’ faces compared with upright faces (Valentine, 1988), is used as an investigation tool to explore the behavioral effects in the field of face perception. Inversion disrupts holistic face processing and necessitates feature-based or detailed strategies, for which our brains may have a template for upright but not inverted faces (Freire et al., 2000), resulting in delayed response latencies and/or decreases in recognition accuracy. Reduced FIE (faster response time for inverted faces) has been demonstrated in BDD (Feusner et al., 2010a; Jefferies et al., 2012) which could be attributed to similar visual processing strategies for upright and inverted faces in BDD individuals, suggesting an aberrant propensity for detailed processing that may have conferred an advantage. Further, reduced FIE on response time was found in BDD individuals compared with healthy controls during face viewing for long duration, but not for short duration (Feusner et al., 2010a). This differential response based on stimuli duration was also replicated in individuals with high body image disturbance using body stimuli (Dhir et al., 2018). Thus, it is possible that those with BDD may have the capacity for normalized configural and holistic processing that is modifiable and potentially malleable during short duration of face viewing.

Moreover, there is evidence that magnocellular pathways in the DVS are tuned to rapid image presentation (Derrington and Lennie, 1984; Schiller et al., 1990; McKeeff et al., 2007; Mullen et al., 2010), thereby facilitating global/holistic visual processing. On the other hand, with higher stimulus frequency/shorter stimulus duration, VVS regions show reduced activation magnitude (Mullen et al., 2010; D’Souza et al., 2011; Gauthier et al., 2012). Given BDD’s phenomenology and evidence from previous studies in BDD, some current and proposed treatment approaches (Beilharz and Rossell, 2017; Johnson et al., 2019; Wilhelm et al., 2019) involve visual modulations or perceptual retraining. Yet, the neural mechanisms underlying aberrant visual information processing and how the neurobiological substrates of potential targets are engaged by different visual modulation approaches are incompletely understood. A mechanistic understanding is critical for the development of, and ability to iteratively refine, effective clinical treatments.

We therefore designed an experiment to test the neurobiological mechanistic effects of a strategy of rapid face stimuli presentation. This strategy required participants to view photos of their own faces (the primary area of appearance concerned in general for most with BDD) for short (125 ms, 250 ms, 500 ms) as well as long (3000 ms) durations. The purpose was to test if shorter durations of face viewing would result in enhanced DVS connectivity, responsible for global/holistic visual processing, and/or suppressed VVS connectivity, responsible for detailed/analytic visual processing.

We employed dynamic effective connectivity (DEC) modeling (Büchel and Friston, 1998) to assess directional connectivity changes from primary visual cortex (V1) to DVS and V1 to VVS. This dynamic connectivity model enabled us to parse out connectivity within the different face stimuli presentation durations. The primary goal was to investigate the effects of rapid face presentation on DVS and VVS connectivity during own-face viewing in those with BDD. As an experimental control, we also investigated connectivity in healthy participants. We hypothesized that brief viewing duration would enhance DVS connectivity and suppress VVS connectivity in BDD and controls. We predicted a lesser effect in BDD than controls due to inherently lower responsiveness of the DVS (Feusner et al., 2007, 2010b,^2011^; Li et al., 2015a; Moody et al., 2015, 2020; Morfini et al., 2017).

## Materials and Methods

### Participants

The UCLA Institutional Review Board approved the study. All participants provided informed written consent. Forty-three unmedicated adults with BDD and 35 healthy controls aged 18-40 years were recruited from the community and were enrolled. BDD participants met DSM-5 criteria for BDD, with face concerns. BDD participants could have comorbid depressive or anxiety disorders, since they commonly co-occur (see Supplementary Material 1 for exclusion criteria).

### Clinical Assessments

Eligibility was determined through telephone screening followed by a clinical interview with the study physician (JDF). The Mini International Neuropsychiatric Interview (MINI) and BDD Module (Eisen et al., 2004; Rief et al., 2006) were administered. The Yale-Brown Obsessive-Compulsive Scale Modified for BDD (BDD-YBOCS) (Phillips et al., 1997), Brown Assessment of Beliefs Scale (BABS) (Eisen et al., 1998), Body Image States Scale (BISS) (Cash et al., 2002), Montgomery-Åsberg Depression Rating Scale (MADRS) (Montgomery and Asberg, 1979), and the Hamilton Anxiety Scale (HAMA) (Hamilton, 1959) were administered to assess BDD symptoms, insight, evaluative/affective experiences of appearance, depression, and anxiety, respectively (see Supplementary Material 2 for assessment details).

### Task Paradigm

**FIGURE 1:**
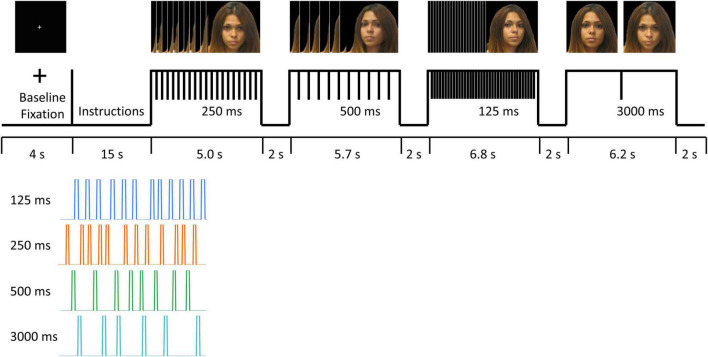
fMRI task paradigm. Informed consent was obtained for publication of the image for the participant in the figure.

Four color photos of participants’ own faces at different, standardized angles were captured before the MRI session. Functional MRI data were acquired while participants viewed photos of their own faces at four different angles for short (125 ms, 250 ms, 500 ms) and long (3000 ms) durations. The blocks of four different stimuli presentation durations were presented for a range of 5.0∼6.8 s, with a brief gap of ∼2 s as an intermittent rest between blocks. The order of different stimuli presentation durations was pseudo randomized, thereby avoiding an order that would have progressively incremental or decremental durations (Figure 1).

### MRI Data Acquisition and Preprocessing

MRI data were acquired on a Siemens Prisma 3T scanner. Data preprocessing was done using fMRIPrep 1.4.0 (Esteban et al., 2019). See Supplementary Materials 3–5 for details of data acquisition and preprocessing, including quality control and motion correction.

### Brain Connectivity Analysis

Fourteen regions-of-interest (ROIs) were derived from the Neurosynth functional meta-analysis in DVS and VVS (Supplementary Figure 1). These included two ROIs in V1 [bilateral calcarine], six ROIs in VVS [bilateral inferior occipital gyrus (IOG), fusiform gyrus (FG), and inferior temporal gyrus (ITG)], and six ROIs in DVS [bilateral superior occipital gyrus (SOG), inferior parietal lobule (IPL), and superior parietal lobule (SPL)]. The ROIs were defined using Neurosynth^1^ with the search terms including “primary visual”, “ventral visual”, “visual stream”, and “dorsal visual” to obtain maps generated with association tests. Blind deconvolution (Wu et al., 2013) was performed on the average timeseries extracted from these ROIs to minimize variability of the hemodynamic response function (HRF) (Wu et al., 2013), thus improving the estimation of effective connectivity (David et al., 2008). DEC, a time-varying measure of directional connectivity between pairs of ROIs, was computed at each time point using time-varying Granger causality (GC) (Büchel and Friston, 1998). The optimization algorithm solving for model parameters in the Kalman filter framework fitted the model to the input timeseries for estimating DEC between ROIs. The model coefficients vary as a function of time, whose lengths were identical to the number of timepoints in the timeseries. Thus, from each DEC timeseries, we were able to extract mean connectivity value corresponding to each of the stimuli durations. Such connectivity estimation for different task stimuli using the same task fMRI time series was made possible by the DEC method. See Supplementary Material 6 for more information. Twelve intra-hemispheric connections (6 per hemisphere) were chosen and divided into 4 categories: 1) VVS_Lower_ *(Calcarine to IOG)*, 2) VVS_Higher_ *(IOG to FG; IOG to ITG)*, 3) DVS_Lower_ *(Calcarine to SOG)*, and 4) DVS_Higher_ *(SOG to IPL; SOG to SPL)*. “Lower” and “Higher” are defined as the connectivity within the occipital cortex (for processing input signals coming from the retina), and the connectivity from the occipital gyri to the temporal or parietal regions (for processing higher-order visual information), respectively. From these twelve connections, the timepoints associated with those trials of viewing faces with different durations were extracted for subsequent statistical analysis.

### Statistical Analysis

Linear mixed models were used to test the primary hypothesis about whether DEC was significantly influenced by experimental factors. Group (BDD or CON), duration (125 ms, 250 ms, 500 ms, or 3,000 ms), connectivity category (DVS_Lower_, DVS_Higher_, VVS_Lower_ or VVS_Higher_), and their interactions were included in the model as fixed factors, with participant ID as random factor. Pairwise comparisons with Bonferroni correction (*p* < 0.05) were performed afterward to determine which factors significantly differed from each other. Further, as a *post hoc* investigation, a ratio of DVS to VVS connectivity was calculated as a fraction of averaged connectivity values in DVS to averaged connectivity values in VVS during different presentation durations. Spearman correlation was used to determine associations between ratios of DVS to VVS connectivity and clinical scores. Statistical tests were done using SPSS and R.

## Results

### Sample Characteristics

Forty-three BDD participants and 35 controls were eligible and scanned. Among these, we excluded three BDD participants and one control due to task paradigm presentation errors. Moreover, we excluded two BDD and five controls’ data due to excessive motion artifacts. Thirty-eight BDD and 29 controls were finally included in the subsequent analyses (Table 1).

**TABLE 1:** Sample characteristics.

	BDD (*n* = 38)	CON (*n* = 29)	Between-group statistics		
			χ^2^	t	*p*-value
**Sex (Male/Female)**	5/33	8/21	1.36		0.24
**Age (Years)**	24.3 ± 6.7	23.1 ± 6.9		0.71	0.48
**Education (Years)**	14.4 ± 1.9	13.5 ± 1.7		2.11	0.04
**Symptoms severity**					
HAMA	10.4 ± 7.2	2.6 ± 2.3		6.22	< 0.001
MADRS	12.9 ± 9.0	1.2 ± 1.5		7.87	< 0.001
BISS	3.7 ± 1.1	6.3 ± 1.1		−9.26	< 0.001
BDD-YBOCS	26.9 ± 4.0	NA			
BABS	15.5 ± 3.8	NA			
**Psychiatric comorbidities**					
Major depressive episode	8				
Persistent depressive disorder (dysthymia)	4				
Panic disorder with agoraphobia	1				
Agoraphobia without history of panic disorder	1				
Social phobia	4				
PTSD	2				
Generalized anxiety disorder	11				
No DSM comorbid disorder	18				

*BDD = body dysmorphic disorder; CON = control; HAMA = Hamilton Anxiety Scale; MADRS = Montgomery-Asberg Depression Rating Scale; BISS = Body Image States Scale; BDD-YBOCS = Yale-Brown Obsessive-Compulsive Scale Modified for BDD; BABS = Brown Assessment of Beliefs Scale; PTSD = Post-traumatic Stress Disorder; χ^2^ = chi-square test; t = independent-samples t-test.*

### Brain Connectivity Patterns

**FIGURE 2:**
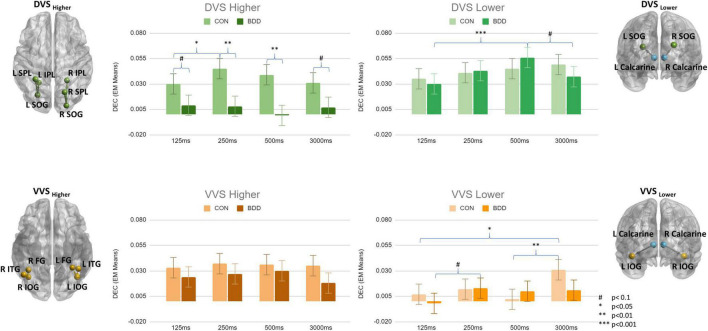
Means of dynamic effective connectivity for the DVS_Higher_, DVS_Lower_, VVS_Higher_ and VVS_Lower_ during different face stimuli presentation durations in the BDD and control groups. The p-values were Bonferroni corrected. The error bars indicate the standard errors.

From tests of fixed effects, there was a significant three-way interaction between group, duration and connectivity category, F[9, 208601] = 2.31, *p* = 0.014. BDD individuals exhibited weaker DEC than controls in DVS_Higher_ during all stimuli presentation durations (125ms: *p* = 0.085; 250 ms: *p* = 0.004; 500 ms: *p* = 0.003; 3000 ms: *p* = 0.064) (Figure 2). There were no significant between-group differences for the other connectivity categories (i.e., DVS_Lower_, VVS_Lower_, and VVS_Higher_). Within-group, from univariate tests, the simple duration effect was significant for VVS_Lower_ in controls, F[3, 208601] = 4.01, *p* = 0.007; they exhibited weaker DEC for VVS_Lower_ during short duration compared to during long duration (125 ms < 3000 ms, p = 0.018; 500 ms < 3000 ms, *p* = 0.007). Further, the simple duration effect was also significant for DVS_Higher_ in controls, F[3, 208601] = 3.48, *p* = 0.015; they showed weaker DEC for DVS_Higher_ during 125 ms duration compared to during 250 ms duration (125 ms < 250 ms, *p* = 0.018). Within the BDD group, the simple duration effect was significant for DVS_Lower_, F[3, 208601] = 5.69, *p* = 0.001, showing the highest DEC value for DVS_Lower_ during 500 ms duration that was greater than during 125 ms and 3,000 ms durations (500 ms > 125 ms, *p* < 0.001; 500 ms > 3000 ms, *p* = 0.071). Although the simple duration effect for VVS_Lower_ did not attain the significance level in BDD (F[3, 208601] = 2.51, *p* = 0.057), the DEC for VVS_Lower_ was observed to be the lowest during 125 ms duration compared to the other three durations (Figure 2). The p-values from pairwise comparisons were Bonferroni corrected.

Since a GC value can be either positive or negative, aggregating all the values could cancel out positive and negative causal effects. As a *post hoc* analysis we separated the values according to their positive/negative sign, and redid the linear mixed models analysis separately for positive and negative values. For positive values, there was no significant three-way interaction between group, duration and connectivity category from tests of fixed effects (F[9, 112012] = 1.01, *p* = 0.431). However, there were significant two-way interactions between group and connectivity category, F[3, 112065] = 24.42, *p* < 0.001, and between duration and connectivity category, F[9, 112012] = 2.10, *p* = 0.026. BDD individuals exhibited weaker DEC than controls in DVS_Higher_ (*p* < 0.001), VVS_Higher_ (*p* = 0.001), and VVS_Lower_ (*p* = 0.005) (Supplementary Figure 2). For negative values, there was significant three-way interaction between group, duration and connectivity category from tests of fixed effects (F[9, 96533] = 3.93, *p* < 0.001). BDD individuals exhibited more negative DEC values than controls in DVS_Lower_ during all stimuli presentation durations (125 ms: *p* = 0.001; 250 ms: *p* = 0.001; 500 ms: *p* = 0.047; 3000 ms: *p* < 0.001) (Supplementary Figure 3). There were no significant between-group differences for the other connectivity categories (i.e., DVS_Higher_, VVS_Lower_, and VVS_Higher_). Within-group, from univariate tests, the simple duration effect was significant for DVS_Lower_ in BDD, F[3, 96532] = 16.78, *p* < 0.001; they exhibited less negative DEC values for DVS_Lower_ during short duration compared to during long duration (125 ms > 3,000 ms, *p* < 0.001; 250 ms > 3,000 ms, *p* < 0.001; 500 ms > 3000 ms, *p* < 0.001). The DEC value for DVS_Lower_ was the least negative during 500 ms duration in BDD that was also less negative than during 125 ms and 250 ms durations (500 ms > 125 ms, *p* < 0.001; 500 ms > 250 ms, *p* = 0.002). For the controls, the simple duration effect from univariate tests was significant for DVS_Higher_ (F[3, 96532] = 4.03, *p* = 0.007) and VVS_Higher_ (F[3, 96533] = 5.00, *p* = 0.002). The DEC value for DVS_Higher_ was the least negative during 250 ms duration that was less negative than during 500 ms and 3,000 ms durations (250 ms > 500 ms, *p* = 0.045; 250 ms > 3000 ms, *p* = 0.013). The DEC value for VVS_Higher_ was the least negative during 3,000 ms duration that was less negative than during 125 ms and 250 ms durations (3000 ms > 125 ms, *p* = 0.001; 3000 ms > 250 ms, *p* = 0.014). The p-values from pairwise comparisons were Bonferroni corrected.

Since the statistically marginal effects could be attributed to the presence of other comorbidities in BDD group, the BDD individuals as a *post hoc* analysis were further divided into two subgroups: BDD with DSM comorbidities (*n* = 20) and BDD without DSM comorbidities (*n* = 18). From tests of fixed effects, there was a significant three-way interaction between group, duration and connectivity category, F[18, 208601] = 2.17, *p* = 0.003. Between-groups, from univariate tests, the simple group effect was significant for DVS_Higher_ during 250 ms (F[2, 90] = 5.16, *p* = 0.008), and 500 ms (F[2, 101] = 5.40, *p* = 0.006) durations. The simple group effect was also significant for VVS_Higher_ during 125 ms (F[2, 84] = 6.86, *p* = 0.002), 500 ms (F[2, 101] = 3.28, *p* = 0.042), and 3,000 ms (F[2, 111] = 4.77, *p* = 0.010) durations. For DVS_Higher_, both BDD with and without DSM comorbidities had on average weaker DEC than controls during all stimuli presentation durations (Supplementary Figure 4). Significant differences were found between controls and BDD with comorbidities during 250 ms (*p* = 0.007), and 500 ms (*p* = 0.006) durations. For VVS_Higher_, BDD with comorbidities had on average weaker DEC than controls, but BDD without comorbidities had on average stronger DEC than controls, during all stimuli presentation durations (Supplementary Figure 4). Significant differences were found between controls and BDD with comorbidities during 125 ms (*p* = 0.038), and 3,000 ms (*p* = 0.031) durations. Significant differences were also found between BDD with and without comorbidities during 125 ms (*p* = 0.001), 500 ms (*p* = 0.040), and 3,000 ms (*p* = 0.019) durations. The *p*-values from pairwise comparisons were Bonferroni corrected.

**FIGURE 3:**
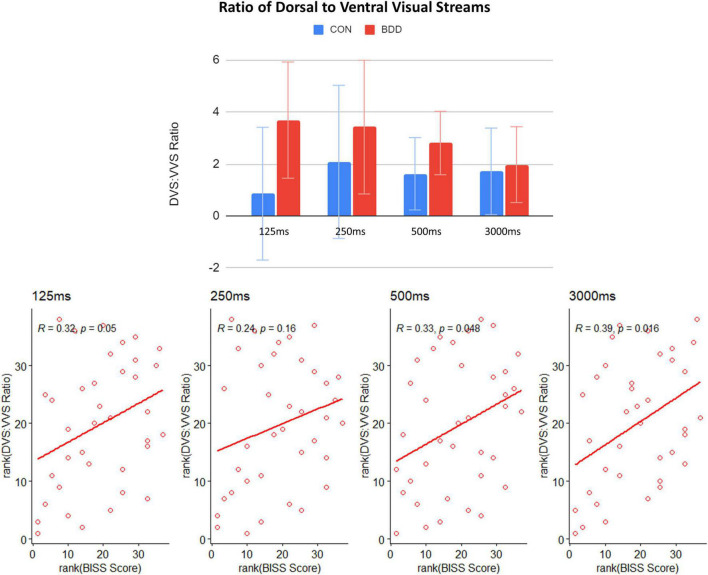
Means of ratios of DVS to VVS connectivity across the four durations in the BDD and control groups, and correlations between the ratios of DVS to VVS connectivity and BISS scores across the BDD participants. The error bars indicate the standard errors.

As an additional *post hoc* investigation, to explore the balance between global and local visual processing, which may influence the ultimate conscious perception of face images and that could be affected by different presentation durations, we calculated the ratio of DVS to VVS connectivity. The ratio was the lowest during the 3,000 ms duration in BDD, and incrementally increased with shorter durations. In BDD, the highest ratio of DVS to VVS connectivity was for the 125 ms duration. For healthy controls, the highest ratio was for the 250 ms duration (Figure 3). Differences between durations were, however, not statistically significant. With associations, there were positive correlations between the ratios of DVS to VVS connectivity and BISS across the four durations in BDD (125 ms: rho = 0.32, *p* = 0.050; 250 ms: rho = 0.24, *p* = 0.160; 500 ms: rho = 0.33, *p* = 0.048; 3,000 ms: rho = 0.39, *p* = 0.016; *p*-values from these Spearman correlation tests were uncorrected). Those with better body image have a higher ratio of DVS to VVS connectivity. This pattern was consistent across the four durations and strongest during the 3,000 ms duration (Figure 3).

## Discussion

The goal of this study was to understand the effects of face stimuli duration on brain connectivity in those with BDD to provide a mechanistic understanding needed to design novel perceptual retraining treatments. We specifically investigated how dynamic effective brain connectivity in visual systems is influenced by different face stimuli presentation durations. While the BDD group demonstrated aberrantly weaker connectivity from occipital to parietal regions in the DVS while viewing their faces across presentation durations, there was no direct evidence in those with BDD that rapid face presentation using the parameters tested in this study effectively enhanced DVS connectivity or suppressed VVS connectivity. A *post hoc* analysis suggested that the ratio of DVS to VVS connectivity incrementally increased, on average, in BDD when the stimuli duration became shorter, although differences among durations were statistically non-significant. The within-group results in healthy controls demonstrate as a proof-of-concept that rapid face presentation could potentially suppress VVS connectivity when stimuli are presented for short durations. Yet, whether, and to what degree, this could occur in those with BDD remains to be determined and likely would necessitate modification of these parameters. These results have implications for future translational research involving perceptual retraining in BDD.

Considering only negative dynamic connectivity values, BDD individuals exhibited more negative values than controls in DVS connectivity from the calcarine cortex to the superior occipital gyri during all stimuli presentation durations. Furthermore, the BDD individuals exhibited less negative values for this DVS connectivity during short duration compared to during long duration. A negative value represents negative causality (i.e., increase in BOLD response of the source timeseries suppresses BOLD response of the target timeseries, and vice versa) (Rangaprakash et al., 2018). These results suggest that BDD individuals demonstrate more suppression of DVS connectivity compared with controls, and this suppression may be reduced with rapid, short presentation durations.

The within-group results in the healthy controls (considering both positive and negative connectivity values) provide promise that rapid face presentation could potentially suppress VVS connectivity with rapid, short presentation durations. This is consistent with previous evidence that the VVS system, responsible for detailed visual processing, decreases activation magnitude with higher stimuli frequency/shorter stimulus duration (Mullen et al., 2010; D’Souza et al., 2011; Gauthier et al., 2012). While the duration effect for the VVS connectivity was observed only at trend level in BDD (*p* = 0.057), the DEC value from the calcarine cortex to the inferior occipital gyri was the lowest during 125 ms duration compared to the other three durations. This pattern thus suggests the potential of suppressing the VVS connectivity in BDD during rapid face presentation with very short stimuli duration; yet the visual systems in those with BDD may be more resistant to immediate effects of this strategy of perceptual modulation. For greater magnitude effects to occur in BDD, different parameters such as a higher number of stimuli and/or repeated sets of stimuli may be necessary, which will be explored in future studies.

Although rapid, short-duration stimuli did not significantly increase DVS connectivity in BDD as hypothesized, it did result in (non-significant) increases in the ratio of DVS to VVS connectivity, providing an early possible signal that, BDD individuals may increasingly engage the DVS system relative to the VVS system during rapid face presentation. Further, better body image self-evaluation (BISS scores) was associated with a higher ratio of DVS to VVS connectivity in BDD for all four durations. These observed neural-behavioral phenotype associations could potentially point to the clinical relevance of this DVS to VVS ratio in BDD. Importantly, the ultimate percept that is consciously experienced may depend less on the individual contributions from global processing or local processing, but the balance (and integration) of the two, which has been found at least, to be important for face recognition (Cheng et al., 2018). These results suggest the possibility that, with more robust design parameters, rapid face presentation could potentially mechanistically increase the ratio of global to local processing in BDD. However, it is important to note that there was wide variability in this ratio across both BDD and healthy control participants. This likely accounted for non-significant differences among different durations and between groups. This warrants explorations in future, larger studies with different parameters and/or identification of subgroups for whom this effect may be more consistent.

Another important finding, although not the primary focus of this study, was that the BDD group had weaker connectivity from occipital to parietal regions in the DVS when viewing their faces compared with controls, which was observed across all face stimuli presentation durations. This pattern is consistent with previous studies in BDD that demonstrated hypoactivity in the DVS regions when viewing low spatial frequency images (Feusner et al., 2007, 2010b,^2011^; Li et al., 2015a,b), and weaker connectivity in parietal network during a body-viewing task (Moody et al., 2020), compared to controls. Further, this pattern has clinical relevance, as demonstrated in our previous study in which those with more severe BDD symptoms had weaker DVS connectivity (Wong et al., 2021). The observation that rapid, short duration stimuli did not significantly increase DVS connectivity strength suggests that this did not have a robust effect of “correcting” this abnormality. It is important to note, however, that for many current treatments for psychiatric disorders, mechanisms of effective symptom improvement do not necessarily require direct, immediate correction of an underlying abnormality (e.g., see Rauch et al., 2006; Moody et al., 2017).

Moreover, from a *post hoc* analysis in which we divided the BDD individuals into two subgroups – BDD with and without DSM comorbidities – we found that both groups had on average weaker DVS connectivity from occipital to parietal regions compared with controls during all stimuli presentation durations. This pattern is consistent with the above results revealed from the two-group comparisons (BDD vs. controls). However, for VVS connectivity from occipital to temporal regions, BDD with comorbidities had on average weaker VVS connectivity than controls, while BDD without comorbidities had on average stronger VVS connectivity than controls, during all stimuli presentation durations. Those with BDD often develop comorbid depressive or anxiety disorders due to the long-term preoccupations with misperceptions of one’s physical appearance and resultant reduced quality of life and ability to function. It is possible that many of those with BDD with comorbidities (particularly anxiety and depressive disorders, which were the most common), in efforts to avoid triggers of negative affective states, might be more likely to try to avoid viewing details of their (perceived) appearance defects, e.g., by avoiding mirror viewing or avoiding getting very close to a mirror. This avoidant pattern, over time, might have an effect of reducing the VVS connectivity responsible for detailed visual processing. On the contrary, those with BDD without comorbidities might be more likely to try to pay attention to areas of concern – a “focused” pattern – as their negative affective states might not be as strong and overridden by their desire to be vigilant to detecting these appearance features in order to find ways to fix or change them. Over time, this might result in enhancement of VVS connectivity. In sum, these psychiatric comorbidities in BDD could lead to a different aberrant visual scanning behaviors, accounting for the differences in these observed visual processing brain connectivity abnormalities. This speculative explanation, however, needs to be directly tested in future studies that more specifically characterize these behaviors than in the current study.

There are several potential translational implications of the findings of this study. The important findings that could influence next-step studies of novel perceptual retraining strategies are that the visual systems in BDD, compared with controls, may be more resistant to the effects of rapid face presentation and thus require different parameters such as greater number of trials and/or repeated sessions. Further, negative DEC connectivity may be the most sensitive marker of the effects of this intervention. If negative connectivity is associated with measured changes in visual perception (being tested in an ongoing study) this element of DEC thus could potentially serve as an important treatment biomarker. If so, the parameters in this study could potentially be used, as they demonstrated significant effects on reducing suppression of DVS connectivity.

There are several limitations to consider. The study population underrepresents the proportion of males with BDD – 13.2% in the current study whereas the figure is closer to about 45% in the general population (Koran et al., 2008; Taqui et al., 2008); thus, findings may not generalize. Another limitation is that we did not assess participants’ emotional states during face viewing (in the interest of not interrupting natural processes involved in face viewing that might be disrupted by self-reflection). Future studies could explore the contribution to visual processing from emotional arousal (Bohon et al., 2012), which could be measured physiologically, and/or using amygdala activation, which might itself change as a result of shorter or longer viewing durations. Further, the study sample size did not permit investigations of subgroups whose visual system connectivity may be more or less responsive to these experimental procedures.

In conclusion, rapid, short-duration face presentation using the parameters tested in this study, although reducing VVS connectivity in healthy controls, did not significantly enhance DVS connectivity or reduce VVS connectivity in those with BDD. The DVS to VVS ratio, representing the balance between global and local processing, showed an early signal for incremental increases corresponding with shorter viewing durations in BDD. Although not as immediately responsive within BDD individuals as in healthy controls, these results nevertheless provide promise that a rapid face presentation strategy, albeit with different parameters, might reduce connectivity in visual systems responsible for local/detailed visual processing. The results from this study provide important mechanistic details about parameters that may need to be modified in order to enact greater magnitude of effects in BDD, as their visual systems may be more resistant to immediate effects of this strategy of perceptual modulation.

## Data Availability Statement

The raw data supporting the conclusions of this article will be made available by the authors, without undue reservation.

## Ethics Statement

The studies involving human participants were reviewed and approved by the UCLA Institutional Review Board. The patients/participants provided their written informed consent to participate in this study. Written informed consent was obtained from the individual(s) for the publication of any potentially identifiable images or data included in this article.

## Author Contributions

WW: methodology, data analysis, investigation, visualization, writing – original draft, reviewing and editing. DR: data acquisition, conceptualization, software, methodology, data analysis, writing – reviewing and editing. TM: data acquisition, data analysis, writing – reviewing and editing. JF: funding acquisition, resources, project administration, conceptualization, experimental design, clinical assessment, data acquisition, methodology, investigation, writing – reviewing and editing, supervision. All authors contributed to the article and approved the submitted version.

## Conflict of Interest

The authors declare that the research was conducted in the absence of any commercial or financial relationships that could be construed as a potential conflict of interest.

## Publisher’s Note

All claims expressed in this article are solely those of the authors and do not necessarily represent those of their affiliated organizations, or those of the publisher, the editors and the reviewers. Any product that may be evaluated in this article, or claim that may be made by its manufacturer, is not guaranteed or endorsed by the publisher.

## Footnotes

1
https://neurosynth.org/
