# Supplementary material for: Dynamic Effective Connectivity Patterns During Rapid Face Stimuli Presentation in Body Dysmorphic Disorder
Source: Front Neurosci. 2022 May 24;16:890424. doi: 10.3389/fnins.2022.890424 (PMC9172595; doi:10.3389/fnins.2022.890424)
Supplement: Supplementary file 1 [file Data_Sheet_1.docx]

**Supplementary Material**

Dynamic effective connectivity patterns during rapid face stimuli presentation in body dysmorphic disorder

**Supplementary Methods**

1. Exclusion criteria
2. Clinical assessments
3. Image acquisition
4. Anatomical data preprocessing
5. Functional data preprocessing
6. Brain connectivity analysis

**Supplementary Figure**

**Figure S1** Locations of the 14 spherical ROIs used for dynamic effective connectivity analysis, overlaid on a brain surface with lateral and ventral views.

**Figure S2** Means of positive dynamic effective connectivity values for the DVS_Higher_, DVS_Lower_, VVS_Higher_ and VVS_Lower_ in the BDD and control groups.

**Figure S3** Means of negative dynamic effective connectivity values for the DVS_Higher_, DVS_Lower_, VVS_Higher_ and VVS_Lower_ during different face stimuli presentation durations in the BDD and control groups.

**Figure S4** Means of dynamic effective connectivity values for the DVS_Higher_, DVS_Lower_, VVS_Higher_ and VVS_Lower_ during different face stimuli presentation durations in the controls and the BDD with/without DSM comorbidities.

**Supplementary References**

1. **Exclusion criteria**

Healthy controls were excluded if they had any current DSM-5 disorder or lifetime bipolar disorder or psychosis. Other exclusion criteria that applied to all participants included psychiatric medications, suicidality, self-injurious behavior, lifetime neurological disorder, current pregnancy, any medical illness that could affect cerebral metabolism, or current treatment with cognitive-behavioral therapy.

1. **Clinical assessments**

***Mini International Neuropsychiatric Interview (MINI):*** The MINI 7.0.0 is a short, structured diagnostic interview developed by psychiatrists and clinicians for DSM-5 and ICD-10 psychiatric disorders. With an administration time of ~15 minutes, the MINI is the structured psychiatric interview of choice for psychiatric evaluation and outcome tracking in clinical psychopharmacology trials and epidemiological studies. The MINI is the most widely used psychiatric structured diagnostic interview instrument in the world, employed by mental health professionals and health organizations in more than 100 countries.

***BDD Diagnostic Module (BDD-DM):*** The BDD-DM is a brief semi-structured diagnostic interview for BDD.

***Yale-Brown Obsessive-Compulsive Scale Modified for BDD (BDD-YBOCS):*** The BDD-YBOCS is a 12-item semi-structured, clinician-rated measure of current BDD severity used in many BDD studies. The first 5 items assess obsessional preoccupations about perceived appearance defects (time preoccupied, interference in functioning and distress due to perceived appearance defects, resistance against preoccupations, and control over preoccupations). Items 6-10 assess BDD-related repetitive behaviors and are similar to items 1-5 (time spent performing the behaviors, interference in functioning due to the behaviors, distress experienced if the behaviors are prevented, and resistance of and control over the behaviors). Item 11 assesses insight into appearance beliefs, and item 12 assesses avoidance because of BDD symptoms. Scores for each item range from 0 (no symptoms) to 4 (extreme symptoms); the total score ranges from 0 to 48, with higher scores reflecting more severe symptoms.

***Brown Assessment of Beliefs Scale (BABS):*** The BABS is a 7-item semi-structured rater-administered scale that assesses insight/delusionality both dimensionally and categorically. BABS items assess the person’s conviction that their belief is accurate, perception of others’ views of the belief, explanation for any difference between the person’s and others’ views of the belief, whether the person could be convinced that the belief is wrong, attempts to disprove the belief, insight, and ideas/delusions of reference related to the belief. The first 6 items are summed to create a total score that ranges from 0 to 24; higher scores indicate poorer insight. Item 7 is not included in the total score, because referential thinking is characteristic of some but not all disorders.

***Body Image States Scale (BISS):*** The BISS is a 6-item questionnaire to rate the current body experience: 1) dissatisfaction-satisfaction with one’s overall physical appearance, 2) dissatisfaction-satisfaction with one’s body size and shape, 3) dissatisfaction-satisfaction with one’s weight, 4) feelings of physical attractiveness-unattractiveness, 5) current feelings about one’s looks relative to how one usually feels, and 6) evaluation of one’s appearance relative to how the average person looks. Responses to each item are based on 9-point, bipolar, Likert-type scales, semantically anchored at each point. The participants were instructed to respond how they felt at that very moment. Higher BISS scores indicate more favorable body image states.

***Montgomery-Åsberg Depression Rating Scale (MADRS):*** The MADRS is a 10-item diagnostic questionnaire which psychiatrists used to measure the severity of depressive episodes. The questionnaire includes questions on the following symptoms: 1) apparent sadness, 2) reported sadness, 3) inner tension, 4) reduced sleep, 5) reduced appetite, 6) concentration difficulties, 7) lassitude, 8) inability to feel, 9) pessimistic thoughts, and 10) suicidal thoughts. Higher score indicates more severe depression, and each item yields a score of 0 to 6. The overall score ranges from 0 to 60.

***Hamilton Anxiety Scale (HAMA):*** The HAMA is a 14-item psychological questionnaire used by clinicians to rate the severity of a patient’s anxiety. Each of the 14 items is defined by a series of symptoms, and measures both psychic anxiety (mental agitation and psychological distress) and somatic anxiety (physical complaints related to anxiety). Each item is rated on a scale of 0 (not present) to 4 (severe), with a total score range of 0-56.

1. **Image acquisition**

A 3T Siemens Prisma scanner was used to obtain the MR images. A T1-weighted structural image was acquired using ultrafast gradient echo sequence for anatomical reference (TR/TE: 2300/2.27 ms; flip angle: 8°; 256 x 256 matrix; voxel size: 1 x 0.977 x 0.977 mm^3^; 192 slices). Functional images were acquired using a T2*-weighted echo planar imaging (EPI) gradient-echo pulse sequence (TR/TE: 1000/33 ms; flip angle: 60°; 104 x 104 matrix; voxel size: 2 mm^3^; 60 slices; 336 volumes per run).

1. **Anatomical data preprocessing**

The T1-weighted (T1w) image was corrected for intensity non-uniformity (INU) (1) with ANTs 2.2.0 (2), and used as T1w-reference. The T1w-reference was skull-stripped, and brain tissue segmentation of cerebrospinal fluid (CSF), white-matter (WM) and gray-matter (GM) was performed on the brain-extracted T1w using FSL 5.0.9 (3). Brain surfaces were reconstructed using FreeSurfer 6.0.1 (4). Volume-based spatial normalization to standard space was performed through nonlinear registration with ANTs 2.2.0, using brain-extracted versions of both T1w reference and the T1w template (MNI152NLin2009cAsym) (5).

1. **Functional data preprocessing**

For each of the 3 fMRI runs per subject, the following preprocessing was performed. A deformation field to correct for susceptibility distortions was estimated based on fMRIPrep’s fieldmap-less approach. The deformation field is that resulting from co-registering the BOLD reference to the same-subject T1w-reference with its intensity inverted (6). Based on the estimated susceptibility distortion, an unwarped BOLD reference was calculated, and was co-registered to the T1w reference using FreeSurfer 6.0.1 which implements boundary-based registration (7). Co-registration was configured with nine degrees of freedom to account for distortions remaining in the BOLD reference. Head-motion parameters with respect to the BOLD reference were estimated before any spatiotemporal filtering using FSL 5.0.9 (8). The BOLD timeseries with slice-timing correction were resampled onto their native space by applying a single, composite transform to correct for head-motion and susceptibility distortions. The BOLD timeseries were resampled into standard MNI space, generating the spatially-normalized, preprocessed BOLD runs. Automatic removal of motion artifacts using independent component analysis (ICA-AROMA) (9) was performed on the preprocessed BOLD timeseries on MNI space after removal of non-steady state volumes and spatial smoothing with an isotropic, Gaussian kernel of 6mm FWHM. Corresponding “non-aggressively” denoised runs were produced after such smoothing. Several confounding timeseries were calculated based on the preprocessed BOLD: framewise displacement (FD), DVARS and three region-wise global signals. FD and DVARS were calculated for each run (10). The three global signals were extracted within the CSF, the WM, and the whole-brain masks. A set of physiological regressors were also extracted to allow for component-based noise correction (CompCor) (11). Principal components were estimated after high-pass filtering the preprocessed BOLD timeseries for the two CompCor variants: temporal (tCompCor) and anatomical (aCompCor). tCompCor components were calculated from the top 5% variable voxels within a mask covering the subcortical regions. aCompCor components were calculated within the intersection of the aforementioned mask and the union of CSF and WM masks calculated in T1w space. Components were also calculated separately within the WM and CSF masks. For each CompCor decomposition, the k components with the largest singular values were retained, such that the retained components’ timeseries were sufficient to explain 50% of variance across the nuisance mask. The remaining components were dropped from consideration. Frames that exceeded a threshold of 0.5 mm FD or 1.5 standardized DVARS were annotated as motion outliers.

1. **Brain connectivity analysis**

A similar analysis strategy has been adopted in our previous study for exploring dynamic connectivity for fear processing in BDD and Anorexia Nervosa (12). We used dynamic effective connectivity (DEC) in this study because of its ability to estimate causal connectivity across time with a precision of each time point, which helped us capture connectivity only within task blocks of interest. DEC was estimated in a Kalman filter based dynamic multivariate vector autoregressive model (dMVAR) framework that is based on the concept of Granger causality (GC). Simulations (13,14) as well as experimental results from electrophysiology and optogenetics (15–18) have demonstrated the ability of GC in estimating fMRI effective connectivity, when latent neural time series data are used after HRF deconvolution (as done in this study).

FMRI is an indirect measure of neural activity corrupted by blood hemodynamics that can alter the relative phase between fMRI time series without having any neural delays between the corresponding regions (19,20). Since this phenomenon can confound fMRI connectivity estimates (21), deconvolution was performed. Additionally, deconvolution has also been found to improve GC estimation (16,17,22). A blind-deconvolution technique developed by Wu et al. (23) was used, which is a data-driven technique based on the point process model (24). It identifies neural events in fMRI data as point processes based on relative intensities and estimates the best fit double-gamma HRF in a least squares sense. Latent neuronal time series is then estimated using Wiener deconvolution. Several studies have employed this technique, e.g., (25–27). The deconvolution was performed using the rsHRF toolbox implemented in SPM12 (<https://github.com/compneuro-da/rsHRF>).

DEC was computed at each time point using Kalman-filter based time-varying GC (28). The basic concept is that if past values of a timeseries can forecast the future values of another timeseries, a causal influence from the former to the latter is inferred. A GC value of 0 represents no causal relationship from source to target timeseries, a value of 1 represents strong positive causality (i.e. increase in BOLD response of the source enhances BOLD response of the target, and vice versa), and a value of -1 represents strong negative causality (i.e. increase in BOLD response of the source suppresses BOLD response of the target, and vice versa) (29). The deconvolved timeseries were fitted into a dMVAR model for estimating DEC between ROIs, as in prior studies (30,31). Further details and underlying mathematics are elaborated in (29,32). The dMVAR model coefficients vary as a function of time, whose lengths were identical to the number of timepoints in the timeseries. That is, a DEC value was obtained for every timepoint for every connection. Using this, we obtained the desired block-specific connectivity values and aggregated them to represent the corresponding connection (33). The timepoints associated with those trials of viewing faces with different durations were extracted for subsequent statistical analysis.

**
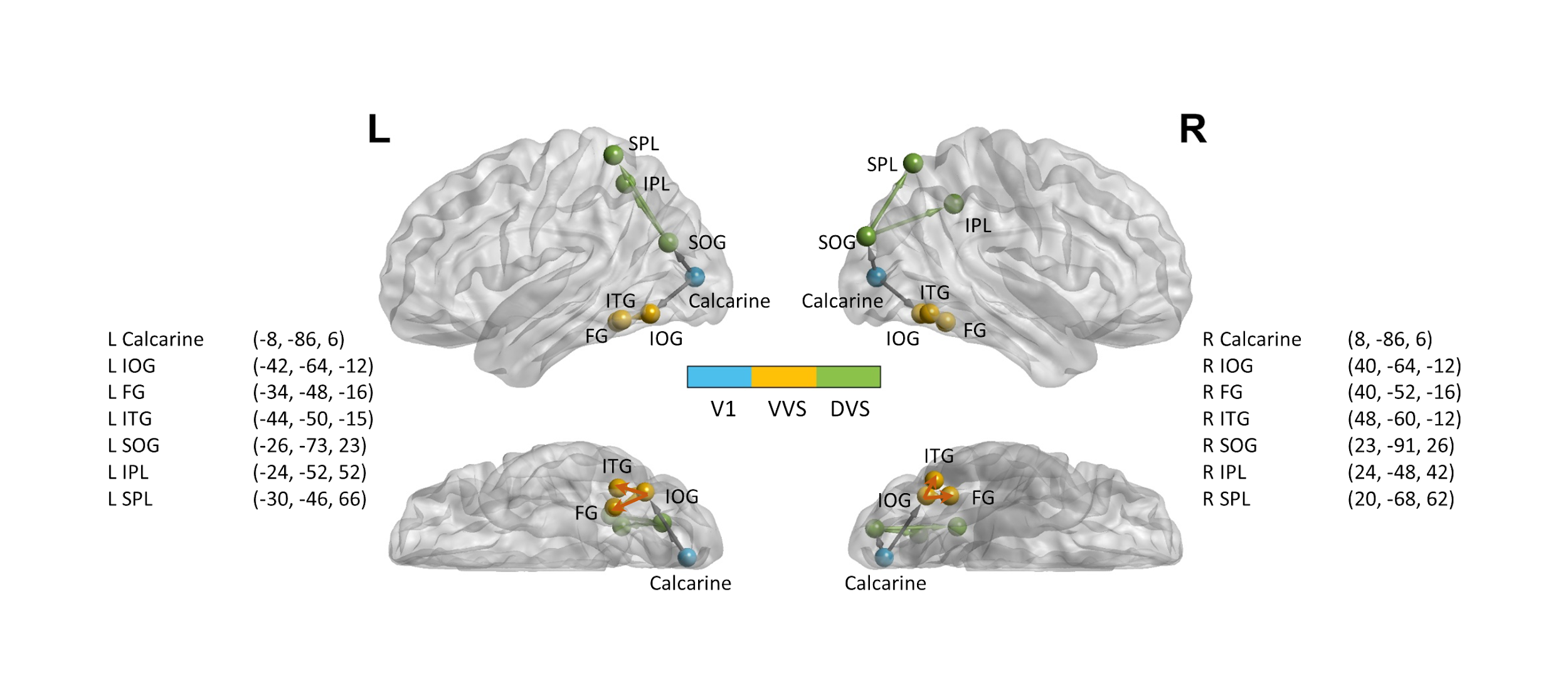
**

**Figure S1** Locations of the 14 spherical ROIs used for dynamic effective connectivity analysis, overlaid on a brain surface with lateral and ventral views. These included 2 ROIs in V1 [bilateral calcarine], 6 ROIs in VVS [bilateral inferior occipital gyrus (IOG), fusiform gyrus (FG), and inferior temporal gyrus (ITG)], and 6 ROIs in DVS [bilateral superior occipital gyrus (SOG), inferior parietal lobule (IPL), and superior parietal lobule (SPL)]. The nomenclature is based on Eickhoff-Zilles macro labels from N27, implemented in AFNI. All spheres had a radius of 5 mm and the center-of-mass coordinates obtained from the clusters are x, y and z in the MNI space. This figure was prepared using BrainNet Viewer (34).

**Figure S2** Means of positive dynamic effective connectivity values for the DVS_Higher_, DVS_Lower_, VVS_Higher_ and VVS_Lower_ in the BDD and control groups. The p-values were Bonferroni corrected. The error bars indicate the standard errors.

**Figure S3** Means of negative dynamic effective connectivity values for the DVS_Higher_, DVS_Lower_, VVS_Higher_ and VVS_Lower_ during different face stimuli presentation durations in the BDD and control groups. The p-values were Bonferroni corrected. The error bars indicate the standard errors.

**Figure S4** Means of dynamic effective connectivity values for the DVS_Higher_, DVS_Lower_, VVS_Higher_ and VVS_Lower_ during different face stimuli presentation durations in the controls and the BDD with/without DSM comorbidities. The p-values were Bonferroni corrected. The error bars indicate the standard errors.

**References**

1. Tustison NJ, Avants BB, Cook PA, Zheng Y, Egan A, Yushkevich PA, Gee JC (2010): N4ITK: improved N3 bias correction. *IEEE Trans Med Imaging* 29: 1310–1320.

2. Avants B, Epstein C, Grossman M, Gee J (2008): Symmetric diffeomorphic image registration with cross-correlation: Evaluating automated labeling of elderly and neurodegenerative brain. *Medical Image Analysis*, vol. 12. pp 26–41.

3. Zhang Y, Brady M, Smith S (2001): Segmentation of brain MR images through a hidden Markov random field model and the expectation-maximization algorithm. *IEEE Transactions on Medical Imaging*, vol. 20. pp 45–57.

4. Dale AM, Fischl B, Sereno MI (1999): Cortical surface-based analysis. I. Segmentation and surface reconstruction. *Neuroimage* 9: 179–194.

5. Fonov VS, Evans AC, McKinstry RC, Almli CR, Collins DL (2009): Unbiased nonlinear average age-appropriate brain templates from birth to adulthood. *NeuroImage*, vol. 47. p S102.

6. Wang S, Peterson DJ, Gatenby JC, Li W, Grabowski TJ, Madhyastha TM (2017): Evaluation of Field Map and Nonlinear Registration Methods for Correction of Susceptibility Artifacts in Diffusion MRI. *Frontiers in Neuroinformatics*, vol. 11. https://doi.org/10.3389/fninf.2017.00017

7. Greve DN, Fischl B (2009): Accurate and robust brain image alignment using boundary-based registration. *NeuroImage*, vol. 48. pp 63–72.

8. Jenkinson M, Bannister P, Brady M, Smith S (2002): Improved optimization for the robust and accurate linear registration and motion correction of brain images. *Neuroimage* 17: 825–841.

9. Pruim RHR, Mennes M, van Rooij D, Llera A, Buitelaar JK, Beckmann CF (2015): ICA-AROMA: A robust ICA-based strategy for removing motion artifacts from fMRI data. *NeuroImage*, vol. 112. pp 267–277.

10. Power JD, Mitra A, Laumann TO, Snyder AZ, Schlaggar BL, Petersen SE (2014): Methods to detect, characterize, and remove motion artifact in resting state fMRI. *NeuroImage*, vol. 84. pp 320–341.

11. Behzadi Y, Restom K, Liau J, Liu TT (2007): A component based noise correction method (CompCor) for BOLD and perfusion based fMRI. *NeuroImage*, vol. 37. pp 90–101.

12. Rangaprakash D, Bohon C, Lawrence KE, Moody T, Morfini F, Khalsa SS, *et al.* (2018): Aberrant dynamic connectivity for fear processing in anorexia nervosa and body dysmorphic disorder. *Front Psychiatry* 9: 273.

13. Wen X, Rangarajan G, Ding M (2013): Is Granger causality a viable technique for analyzing fMRI data? *PLoS One* 8: e67428.

14. Ryali S, Supekar K, Chen T, Menon V (2011): Multivariate dynamical systems models for estimating causal interactions in fMRI. *Neuroimage* 54: 807–823.

15. Katwal SB, Gore JC, Gatenby JC, Rogers BP (2013): Measuring relative timings of brain activities using fMRI. *Neuroimage* 66: 436–448.

16. David O, Guillemain I, Saillet S, Reyt S, Deransart C, Segebarth C, Depaulis A (2008): Identifying neural drivers with functional MRI: an electrophysiological validation. *PLoS Biol* 6: 2683–2697.

17. Ryali S, Shih Y-YI, Chen T, Kochalka J, Albaugh D, Fang Z, *et al.* (2016): Combining optogenetic stimulation and fMRI to validate a multivariate dynamical systems model for estimating causal brain interactions. *Neuroimage* 132: 398–405.

18. Wang Y, Katwal S, Rogers B, Gore J, Deshpande G (2017): Experimental validation of dynamic Granger causality for inferring stimulus-evoked sub-100 ms timing differences from fMRI. *IEEE Trans Neural Syst Rehabil Eng* 25: 539–546.

19. Aguirre GK, Zarahn E, D’Esposito M (1998): The variability of human BOLD hemodynamic responses. *Neuroimage* 7: S574.

20. Handwerker DA, Ollinger JM, D’Esposito M (2004): Variation of BOLD hemodynamic responses across subjects and brain regions and their effects on statistical analyses. *Neuroimage* 21: 1639–1651.

21. Rangaprakash D, Wu G-R, Marinazzo D, Hu X, Deshpande G (2018): Hemodynamic response function (HRF) variability confounds resting-state fMRI functional connectivity. *Magn Reson Med* 80: 1697–1713.

22. Ryali S, Chen T, Supekar K, Menon V (2012): Estimation of functional connectivity in fMRI data using stability selection-based sparse partial correlation with elastic net penalty. *Neuroimage* 59: 3852–3861.

23. Wu G-R, Liao W, Stramaglia S, Ding J-R, Chen H, Marinazzo D (2013): A blind deconvolution approach to recover effective connectivity brain networks from resting state fMRI data. *Med Image Anal* 17: 365–374.

24. Saad ZS, Gotts SJ, Murphy K, Chen G, Jo HJ, Martin A, Cox RW (2012): Trouble at rest: how correlation patterns and group differences become distorted after global signal regression. *Brain Connect* 2: 25–32.

25. Boly M, Sasai S, Gosseries O, Oizumi M, Casali A, Massimini M, Tononi G (2015): Stimulus set meaningfulness and neurophysiological differentiation: a functional magnetic resonance imaging study. *PLoS One* 10: e0125337.

26. Amico E, Gomez F, Di Perri C, Vanhaudenhuyse A, Lesenfants D, Boveroux P, *et al.* (2014): Posterior cingulate cortex-related co-activation patterns: a resting state FMRI study in propofol-induced loss of consciousness. *PLoS One* 9: e100012.

27. Lamichhane B, Adhikari BM, Brosnan SF, Dhamala M (2014): The neural basis of perceived unfairness in economic exchanges. *Brain Connect* 4: 619–630.

28. Büchel C, Friston KJ (1998): Dynamic changes in effective connectivity characterized by variable parameter regression and Kalman filtering. *Hum Brain Mapp* 6: 403–408.

29. Rangaprakash D, Dretsch MN, Venkataraman A, Katz JS, Denney TS Jr, Deshpande G (2018): Identifying disease foci from static and dynamic effective connectivity networks: Illustration in soldiers with trauma. *Hum Brain Mapp* 39: 264–287.

30. Hutcheson NL, Sreenivasan KR, Deshpande G, Reid MA, Hadley J, White DM, *et al.* (2015): Effective connectivity during episodic memory retrieval in schizophrenia participants before and after antipsychotic medication. *Hum Brain Mapp* 36: 1442–1457.

31. Feng C, Deshpande G, Liu C, Gu R, Luo Y-J, Krueger F (2016): Diffusion of responsibility attenuates altruistic punishment: A functional magnetic resonance imaging effective connectivity study. *Hum Brain Mapp* 37: 663–677.

32. Rangaprakash D, Dretsch MN, Katz JS, Denney TS Jr, Deshpande G (2019): Dynamics of segregation and integration in directional brain networks: Illustration in soldiers with PTSD and neurotrauma. *Front Neurosci* 13: 803.

33. Sathian K, Deshpande G, Stilla R (2013): Neural changes with tactile learning reflect decision-level reweighting of perceptual readout. *J Neurosci* 33: 5387–5398.

34. Xia M, Wang J, He Y (2013): BrainNet Viewer: a network visualization tool for human brain connectomics. *PLoS One* 8: e68910.
